# ‘I tested positive, so do I have the condition or not?’

**DOI:** 10.1007/s12471-021-01553-y

**Published:** 2021-02-18

**Authors:** R. Delewi, Y. M. Pinto

**Affiliations:** Department of Cardiology, Amsterdam University Medical Centre, location Academic Medical Centre, University of Amsterdam, Amsterdam, The Netherlands

Imagine a 55-year-old woman who is scheduled for gallbladder surgery. She opens her own electronic patient file to check on the latest test results and sees that the routine electrocardiogram (ECG) mentions: ‘signs of a prior inferior myocardial infarction’. Understandably, this comes as a shock for someone without any prior cardiac complaint. How should we interpret these unexpected findings?

In this issue of the *Netherlands Heart Journal*, Lobeek et al. describe an analysis that may be—partly—of help to our patient and is therefore highly relevant [1]. The authors assessed the value of ECG to detect a prior myocardial infarction (MI) by retrospectively studying over a 1000 patients who had undergone cardiac magnetic resonance (CMR) imaging with late gadolinium enhancement (LGE) and for whom a recent ECG was available. An important point to note is that our imaginary patient is not likely to be included in this study population: all these subjects had some reason to undergo CMR imaging in addition to an ECG, which she does not.

Notwithstanding, the sobering outcome of the analysis by Lobeek and colleagues does shed light on how to deal with unexpected findings. Their analysis revealed only a moderate negative predictive value of a normal ECG: absence of signs of a previous MI on ECG was confirmed by the same absence on CMR with LGE in 84% of cases. However, in those cases where CMR with LGE revealed a pattern of a prior MI, this was only detected in around 40% of the patients by ECG. How do we interpret these numbers? What does it mean for our patient—again realising the caveat that she would not have been included in this analysis?

As with all diagnostic tests, one needs to take the prior risk of the disease into account, as this massively drives the actual chances of the test to add significantly to the risk. The short summary is that most diagnostic tests that we are so used to in practice, actually add little information when judged in isolation. When the prior chance of having the disease is low, only extraordinarily sensitive *and* specific tests are able to add considerably to the chance of finding the disease. In that light, a routine ECG performs very poorly as a single measure to detect an old MI, as does a single abnormality on clinical examination. Often, mainly when combined, do such tests add valuable information for the patient. The essential step is to obtain a good estimate of the *a priori* chance to get a more reliable estimate of the outcome of the test.

Let us take a look at how this pans out in the study by Lobeek et al. Suppose we line up a thousand women of 55 years old scheduled for surgery. When we grossly simplify their data in Table 2, the overall rate of finding a true prior MI is around 20% (i.e. 200 of 1000 cases). With a sensitivity of 40%, 80 of these 200 ECGs would show a prior MI, while given a specificity of 85%, 15% of the ECGs (i.e. 150 patients) would show false-positive signs. In total, 230 (150 + 80) ECGs would display signs of a prior MI. Thus, whereas the chance to have had a prior MI *before* taking an ECG was 20%, an abnormal ECG increases the chance that the patient truly had a prior MI to 35% (80/230).

If this is not sobering enough, it is important to consider that some series report even much lower prevalences, down to 8% in populations showing a prior MI on CMR [2]. In that case, the *a priori* chance of 8% rises to 18% upon finding ‘signs of a prior MI’ on the ECG. In other words, if our patient is in a low-risk category, the data by Lobeek et al. suggest that her actual chance of having had a prior MI is still very low.

In other words, we are all taught that if we hear a car driving by, it is most likely not to be a Ferrari. However, this is not entirely true. What the sound of a nearing car means may depend on our surroundings: if you are enjoying a glass of wine on a warm and sunny afternoon listening to the sounds of the Côte d’Azur and you hear excited paparazzi in the background, the sound of a car may more likely signify the nearing of a Ferrari than of an Opel.
